# Development, optimization, and evaluation of a nanostructured lipid carrier of sesame oil loaded with miconazole for the treatment of oral candidiasis

**DOI:** 10.1080/10717544.2021.2023703

**Published:** 2022-01-11

**Authors:** Khaled M. Hosny, Amal M. Sindi, Sarah Ali, Waleed S. Alharbi, Maher S. Hajjaj, Haitham A. Bukhary, Moutaz Y. Badr, Rayan Y. Mushtaq, Samar S. A. Murshid, Alshaimaa M. Almehmady, Rana B. Bakhaidar, Eman Alfayez, Mallesh Kurakula

**Affiliations:** a Faculty of Pharmacy, Department of Pharmaceutics, King Abdulaziz University, Jeddah, Saudi Arabia; b Faculty of Dentistry, Department of Oral Diagnostic Sciences, King Abdulaziz University, Jeddah, Saudi Arabia; c Faculty of Dentistry, Department of Restorative Dentistry, King Abdulaziz University, Jeddah, Saudi Arabia; d Department of Pharmaceutics, Collage of Pharmacy, Umm Al-Qura University, Makkah, Saudi Arabia; e Department of Pharmaceutics, Collage of clinical pharmacy, Imam Abdulrahman Bin Faisal University, Dammam, Saudi Arabia; f Faculty of Pharmacy, Department of Natural Products and Alternative Medicine, King Abdulaziz University, Jeddah, Saudi Arabia; g Faculty of Dentistry, Department of Oral Biology, King Abdulaziz University, Jeddah, Saudi Arabia; h Department of Biomedical Engineering, The Herff Collage of Engineering, Memphis, TN, USA

**Keywords:** Antifungal, sesame oil, *Candida albicans*, nanostructured lipid carrier, experimental design, ulcer index

## Abstract

*Candida albicans* is the fungus responsible for oral candidiasis, a prevalent disease. The development of antifungal-based delivery systems has always been a major challenge for researchers. This study was designed to develop a nanostructured lipid carrier (NLC) of sesame oil (SO) loaded with miconazole (MZ) that could overcome the solubility problems of MZ and enhance its antifungal activity against oral candidiasis. In the formulation of this study, SO was used as a component of a liquid lipid that showed an improved antifungal effect of MZ. An optimized MZ-loaded NLC of SO (MZ-SO NLC) was used, based on a central composite design-based experimental design; the particle size, dissolution efficiency, and inhibition zone against oral candidiasis were chosen as dependent variables. A software analysis provided an optimized MZ-SO NLC with a particle size of 92 nm, dissolution efficiency of 88%, and inhibition zone of 29 mm. Concurrently, the *ex vivo* permeation rate of the sheep buccal mucosa was shown to be significantly (*p* < .05) higher for MZ-SO NLC (1472 µg/cm^2^) as compared with a marketed MZ formulation (1215 µg/cm^2^) and an aqueous MZ suspension (470 µg/cm^2^). Additionally, an *in vivo* efficacy study in terms of the ulcer index against *C. albicans* found a superior result for the optimized MZ-SO NLC (0.5 ± 0.50) in a treated group of animals. Hence, it can be concluded that MZ, through an optimized NLC of SO, can treat candidiasis effectively by inhibiting the growth of *C. albicans*.

## 1. Introduction

Oral candidiasis (OCA), also known as thrush, is an infection of the oral mucosa and lingua present in superficial tissue (Rençber et al., 2016; Vila et al., 2020). OCA occurred even in ancient civilizations (400 BC), where it was described by Hippocrates in his book *Of the Epidemics* (Patil et al., 2015). In 1839, Langenbeck was the first researcher to reveal the involvement of a fungus in the pathogenesis of OCA. After some misclassifications and confusion in naming during the 1800s and early 1900s, the binomial classification of *Candida albicans* was fully endorsed in 1954 (Patil et al., 2015). *Candida albicans* (CA) is considered a major pathogenic agent responsible for OCA. Based on various clinical findings, it was revealed that CA resides in the oral mucosa, as well as in the gastrointestinal tract and that it proliferates in response to the host immune response (Serrano et al., 2020). The pathogenesis of OCA occurs as CA undergoes a yeast-to-hypha transition and penetrates the epithelia via the formation of filaments. In addition to the yeast-to-hypha transition, endocytosis is another pathological mechanism involving E-cadherin and other adherens junctions. Aspartyl proteinases (SAPs), secreted phospholipases (PLs), and candidalysin are enzymes and toxins secreted by CA and cause damage to mucosal tissues (Serrano et al., 2020). One of the common clinical features of CA infection is the formation of a biofilm, which is made up of β-1,3-glucan, mannans, and β-1,6-glucan and accounts for the exponential growth of pathogens. In the buccal cavity, the dorsal side of the tongue is mostly affected. The disease is diagnosed by invasion into the epithelial lining of the tongue and another region of the buccal cavity (Talapko et al., 2021). Among various risk factors, glandular dysfunction, nutritional deficiency, prolonged use of several drugs, immunological dysfunction, malignancy, use of dental prostheses, and smoking are primarily responsible for OCA. OCA has several deleterious effects on the quality of life of patients. On the one hand, this disease causes tremendous discomfort in eating and drinking, and, on the other hand, it results in coexisting disease that severely impacts psychological and neurobehavioral functions, leading to social withdrawal and a poor quality of life (Talapko et al., 2021).

Among the various pharmacotherapeutic drugs, antifungal drugs, mainly azoles, are the drugs of choice for treating OCA. Azole drugs act via inhibition of lanosterol demethylase and peroxidases, which are responsible for the formation of ergosterol, which causes OCA (Gheorghe et al., 2021). Among various azoles, miconazole (MZ) is extensively used and has shown significant clinical outcomes. However, MZ is a weak acid and poorly aqueous soluble drug and hence it shows lower bioavailability, as well as a limited therapeutic outcome (Gheorghe et al., 2021). Sesame oil (SO) is a commonly used vegetable oil obtained from *Sesamum indicum*. SO has been reported to have potent antioxidant, anti-inflammatory, immunoregulatory, antibacterial, and antiulcer potential in various preclinical studies. SO is a safe essential oil and has been endorsed by the United States Food and Drug Administration for use as an additive (Ogawa et al., 2014). MZ has proven effective as an antifungal drug but has pharmacokinetic limitations, and SO is a naturally occurring oil with proven effectiveness as an antioxidant, anti-inflammatory, and antiulcer agent. However, oils such as SO also suffer from the limitations of low aqueous solubility and instability, and hence, their widespread use is limited in the pharmaceutical industry (Zhang et al., 2016).

To cover the existing limitations of free MZ, an MZ-loaded nanostructured lipid carrier (NLC) of SO appears to be a novel drug delivery system in which a combination of a natural drug and a synthetic drug will exhibit enhanced antifungal effects against OCA (Mendes et al., 2013). NLCs are second-generation lipid-based nanocarrier systems fabricated via the blending of a solid lipid, a liquid, and a surfactant. NLC offers several advantages, such as a high drug-loading capacity, a controlled release pattern, biocompatibility, stability, and safety (Mendes et al., 2013).

It is important to understand that the oral mucosa is highly vascularized, has comparatively lower enzymatic activity, and is devoid of first-pass metabolism. Hence, an NLC-based targeted drug delivery system appears to be an emerging and novel drug delivery system in which the mucosal membrane comes in contact with NLC with an enhanced retention time and a good therapeutic outcome (Santiago et al., 2018). To make an optimized NLC-based nanoformulation, an experimental design was utilized as a statistical tool. This approach simplified the exhaustive process of formulation development, and the obtained statistical design and mathematical equations exhibited the effects of different selected variables on the nanoformulations (Imran et al., 2020).

Thus, in the current study, an SO-based NLC of MZ (MZ-SO NLC) to target CA-induced OCA was developed using an experimental design in which the particle size, dissolution efficiency, and inhibition zone were selected as dependent variables. Further, the morphology, the permeation of the drug, and an *in vivo* ulcer index were determined.

## 2. Materials and methods

### 2.1. Materials

MZ as a pure drug was received as a gift sample from Bayer (Berlin, Germany), and SO was procured from Acros Organics (Carlsbad, California, USA). Various other reagents and chemicals, such as glycerol dibehenate (Compritol), caprylocaproyl polyoxyl-8 glycerides (Labrasol), and propylene glycol monocaprylate (Capryol 90), were obtained from Gattefosse (Saint-Priest, France). Ethanol, chloroform, acetonitrile, and methanol were purchased from Sigma-Aldrich (St. Louis, Missouri, USA). All other chemicals and solvents were of analytical grade.

### 2.2. Experimental design for the selection of an optimized MZ-SO NLC

In this study, a central composite design (CCD)-based experimental design (Design-Expert software v. 13.0.5.0 (Stat-Ease, Inc., Minneapolis, Minnesota, USA), was utilized for the selection of an optimized NLC with the three independent variables, the drug-to-total lipid ratio (*X*_1_), oil-to-solid lipid ratio (*X*_2_), and surfactant concentration (*X*_3_). The particle size (*Y*_1_), dissolution efficiency of MZ (*Y*_2_), and inhibition zone against OCA (*Y*_3_) were chosen as dependent variables (Table 1).

**Table 1.** The uncoded and coded levels of the NLC formulation factors were investigated using the face-centered central composite design.

Factor	Level		
Independent variables	−1	0	+1
X_1_: Total lipid-to-drug ratio	10:1	15:1	20:1
X_2_: Oil-to-solid lipid ratio	1:4	2:4	4:4
X_3_: Surfactant concentration (%)	0.5	1	2
Dependent variables	Constraints		
Y_1_: Particle size	Minimum		
Y_2_: Dissolution efficiency (DE)	Maximum		
Y_3_: Inhibition zone against oral candidiasis (Y3)	Maximum		

### 2.3. Preparation of MZ-SO-loaded NLC

**Figure 1.**
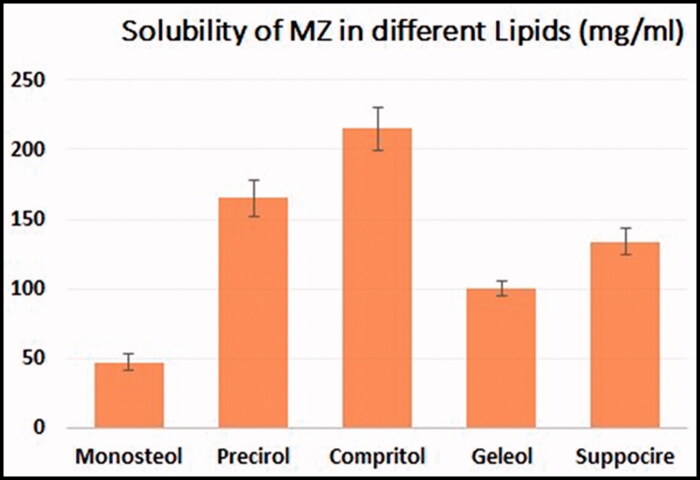
Solubility of MZ in various lipids.

Using a hot homogenization method followed by sonication, different formulations of MZ-SO NLCs were prepared (Mussi et al., 2014). For the preparation, every nanoformulation contained a lipid phase in which predetermined quantities of Compritol, SO, Capryol 90, and MZ (20 mg) were dissolved in a 1:1 mixture of chloroform and methanol. The solid lipid was selected according to a preliminary solubility study for MZ in different types of lipids from which Compritol was selected, data for solubility is presented in Figure 1. The organic solvent mixture was removed completely using a rotary evaporator (R 10 BÜCHI Labortechnik AG, Flawil, Switzerland). The drug’s lipid layer was melted at 65–70 °C. At the same time, an aqueous phase was prepared by dissolving a predetermined amount of Labrasol in distilled water and then warming the mixture at 65–70 °C. Next, a prepared warmed aqueous medium was poured into the lipid phase, and the mixture was homogenized (IKA Homogenizer T18; IKA, Karnataka, India) at a speed of 6000–24,000 rpm for 2 min. The prepared emulsion was sonicated using a probe sonicator (Sonics Vibra-Cell VCX 750; Sonics & Materials, Inc., Newtown, Connecticut, USA) for 1 min, and the fabricated NLCs were kept at 25 °C. For further characterization, the prepared MZ-SO NLCs were lyophilized (Martin Christ GmbH, Osterode am Harz, Germany) at a condenser temperature of −45 °C and pressure of 7 × 10^−2^ mbar. And the encapsulation efficiency was determined for prepared formulation.

### 2.4. Characterization of prepared MZ-SO-loaded NLCs

#### 2.4.1. Particle size

The particle size of prepared nanoformulations was determined by dynamic light scattering using a Zetatrac particle size analyzer (Microtrac, Inc., York, Pennsylvania, USA). For this purpose, every 1 ml of the collected samples was diluted with 20 ml of distilled water separately and agitated for 3 min. Then, 1-ml diluted samples were used for the determination of the particle size (Hosny et al., 2020).

#### 2.4.2. Determination of *in vitro* dissolution efficiency

For the determination of the *in vitro* dissolution efficiency of each prepared formulation, an automated vertical Franz diffusion cell (Hanson Research Microette Plus, Chatsworth, California, USA) was used. An activated Dialysis Tubing Cellulose Membrane (cut-off of 14,000 Da pore size and diffusion area of 1.7 cm^2^) was used as a barrier membrane. The membrane was activated by being first soaked in phosphate-buffered saline (PBS) at a pH of 6.8 at 25 + 0.05 °C for 15 min. Next, 25 ml of PBS contain 0.1% polysorbate 80 was placed in the receptor compartment of the Franz diffusion cell and stirred continuously using a magnetic stirrer as dissolution media which ensures sink condition. Simultaneously, predetermined amounts of lyophilized nanoformulations were evenly distributed in the donor compartment over the membrane. In order to determine the dissolution efficiency, a sample of 2 ml was collected at every predetermined time point (0.5, 1.00, 2.00, 4.00, 6.00, 8.00, 10.00, and 12.00 h) and the MZ concentration was analyzed spectrophotometrically (UV/Visible Spectrophotometer, Jenway 6705, Vernon Hills, Illinois, USA) at 272 nm. The percentage was calculated compared with the quantity of MZ in the NLCs, which was analyzed for every formulation before executing dissolution analysis via an indirect method (Opatha et al., 2020). For this purpose, 4-ml samples were collected from each MZ-SO NLC and centrifuged (3K30 centrifuge, Sigma Centrifuges, SciQuip Ltd., Newtown, Wem, Shropshire, UK) at 20,000 rpm for 60 min. The MZ that was not trapped was analyzed spectrophotometrically from the clear supernatant. Additionally, the amount of entrapped drugs was calculated by dividing the amount of drug-loaded by the amount of theoretical drug loaded. These studies were carried out in replicate for every formulation.

#### 2.4.3. Determination of inhibition zones

For this *in vitro* antifungal study, CA was used as a fungal strain grown in sabouraud dextrose agar medium (SDA).

##### 2.4.3.1. Preparation of inoculum

To obtain the required concentration of CA, three to five colonies were selected and suspended in 2 ml of normal saline and vortexed. First, the 0.5 McFarland Standard was used to adjust the turbidity of the solution. Next, a sterile cotton swab was used to prepare SDA plates to obtain a lawn culture.

##### 2.4.3.2. Disk diffusion method

The disk diffusion method was used for the determination of the antifungal potential of the developed formulation. 5mm of disk filter paper were placed over 100 μl of MZ-SO NLC and inoculated over previously infected dried SDA plates at 24 ± 2 °C for 48 h. The inhibition zone was recorded and compared with that of the 99.9% ethanol (positive control).

#### 2.4.4. Selection of optimized MZ-SO NLC nanoformulation

The CCD-based experimental design software was used to optimize the nanoformulation, and it was performed as per the constraints and goals depicted in Table 1. After the software-based analysis of obtained responses in terms of particle size, dissolution efficiency, and inhibition zone, the optimum nanoformulation was selected. Further, selected optimized MZ-SO NLC was evaluated on various parameters such as ultramicroscopy, permeation, and *in vivo* efficacy.

### 2.5. Ultramorphological analysis of MZ-SO NLC

A transmission electron microscope (TEM) was used to analyze the morphological and structural characteristics of the MZ-SO NLCs (TEM H7500; Hitachi, Japan). For this purpose, initially, samples were diluted 200-fold with double distilled water. Then diluted samples were placed on copper grids supported by Formvar films. The excess samples were drawn off with filter paper. Next, samples were stained in 0.5% phosphotungstic acid solution for 30 sec and observed after drying. The TEM operated at 80 kV, enabling point-to-point resolutions (Iqubal et al., 2021).

### 2.6. *Ex vivo* permeation studies for the optimized MZ-SO NLC

For the *ex vivo* permeation studies of the optimized MZ-SO NLC formulations, 0.5 g of 2% MZ-SO, 2% MZ suspension, and 2% MZ marketed formulation were used. For the *ex vivo* permeation study, oral mucosa was obtained from the local slaughterhouse, immediately after the slaughtering and before scaling. Obtained mucosa was immediately transferred to the laboratory under refrigerator condition. Before packaging the mucosa for transportation to the laboratory, it was moistened with 10 mol L^−1^ HEPES buffer. In the laboratory, mucosa (2 × 2 cm) was isolated from the buccal cavity kept hydrated, until the experiment was conducted using Franz diffusion cell (MicroettePlus) (Matos et al., 2020). 7 ml phosphate buffer was used as a diffusion medium and maintained at a pH of 6.8, containing 0.1% polysorbate 80 to overcome sink limitation and a temperature of 32 ± 2 °C. The magnetic stirrer was fixed at 400–420 rpm, and at the fixed interval of time, samples were removed and analyzed by high-performance liquid chromatography (HPLC) (Hermawan et al., 2017). For this purpose, C_8_ column was used, and the mobile phase contained a mixture of methanol and water (85:15). Samples were analyzed at 220 nm with a 0.80 ml/min flow rate. The rate of drug diffusion across the mucosal membrane was calculated by the cumulative amount of drug diffused (Q) per unit area of time. The *ex vivo* permeation studies help in analyzing various pharmacokinetic factors, such as steady-state flux (Jss), diffusion coefficient (D), enhancement factor (EF), and permeability coefficient (Pc) (Pinto et al., 2020). The amount of MZ diffused into the receptor chamber and the percentage of permeation were calculated using the following equation:

(1) Percentage of permeation = [MZp/MZT] × 100 ……. 

where MZp is the amount of MZ that permeated the receptor chamber and MZT is the initial amount of MZ in the donor chamber.

### 2.7. Determination of *in vivo* efficacy of optimized MZ-so NLC against OCA

The experimental protocol of the research was approved by the Local Institutional Animal Ethics Committee of Beni-Suef University (Approval No. 631-7-21), and the research work was conducted as per the Guide for the Care and Use of Laboratory Animals (NIH publication #85-23, revised in 2011). For the animal study, male albino rats (160–240 g) were used, randomly divided into four groups (*n* = 6), and treated with normal saline (negative control), 2% MZ-SO NLC, 2% MZ marketed gel, or SO alone.

To obtain the required concentration of CA, three to five colonies were selected and suspended in 2 ml of normal saline and vortexed. The colonies were centrifuged to obtain the final concentration of 3 × 10^8^ CFU/ml of the CA. An amount of 0.1 ml of the obtained CA suspension (3 × 10^8^ CFU/ml) was used for the induction of OCA. A cotton swab containing 0.1 ml of CA was rolled twice along the oral cavity of all the experimental animals on days 3, 5, and 7. The MZ-SO NLC, MZ suspension, and marketed MZ gel were applied for the first 3 days and at the end of 3 days (Al-Yahya & Asad, 2016; Mostofa et al., 2017). The mucosal ulceration index was calculated with a score of 0–5, where 0 was the normal architecture of epithelial cells; 1 was the epithelial cells with redness; 2 was ulcers of less than 3 mm, 3 was ulcers of 1–2 mm without hemorrhage, 4 was ulcers of 1–2 mm with hemorrhage, and 5 was ulcers of greater than 2 mm (Alkhalidi et al., 2021). The results were recorded, and the effects of the formulations on the ulcer index were analyzed via a one-way ANOVA test.

### 2.8. Statistical analysis

The obtained results were presented as the mean ± standard deviation (SD), and the results of the different groups were compared statistically via the t-test and one-way ANOVA. A *p*-value of less than .05 was considered a significant difference between the compared groups.

## 3. Results and discussion

### 3.1. Selection of optimized MZ-SO NLC

In order to obtain an optimized formulation, 17 different MZ-SO NLCs were prepared as suggested by the experimental design software with the help of different ingredients such as the total lipid-to-MZ ratio, SO-to-Compritol ratio, and Labrasol concentration, and these ingredients were considered independent variables for the CCD-based experimental design. All the fabricated NLCs were characterized for particle size (Y_1_), dissolution efficiency of MZ (Y_2_), and inhibition zone against OCA (Y_3_) and were chosen as dependent variables (Table 2). Also, the encapsulation efficiency for the prepared formulation was determined and was found to be 96 ± 3%. The significance models were achieved by an ANOVA, in which the results clearly indicated significant model terms as per all three dependable variables and indicated an admissible fit to the articulated model. The impact of every single model term was evaluated using response surface analysis. Different three-dimensional (3D) response surface plots were obtained via the experimental software for particle size, dissolution efficiency, and inhibition zone.

**Table 2.** The obtained particle size from various trials by the experimental central composite design (CCD) for the SO-based NLCs of miconazole (MZ-SO NLCs).

	Factor 1	Factor 2	Factor 3	Response 1	Response 2	Response 3
Run	A: Total lipid-to-MZ ratio	B: SO-to-compritol ratio	C: Labrasol concentration (%)	Particle size (nm)	Dissolution efficiency (%)	Inhibition zone (mm)
1	0	−1	0	270	55	9
2	0	0	0	235	59	17
3	1	1	1	95	64	22
4	1	0	0	255	39	13
5	0	0	0	206	55	18
6	0	0	0	270	57	17
7	−1	−1	1	161	70	16
8	−1	−1	−1	390	43	13
9	0	1	0	100	60	27
10	1	−1	−1	410	36	5
11	−1	1	1	92	88	29
12	1	1	−1	270	41	18
13	−1	1	−1	240	55	19
14	0	0	−1	320	44	13
15	−1	0	0	220	79	19
16	1	−1	1	167	59	13
17	0	0	1	121	71	19

#### 3.1.1. Impacts of independent factors on particle size

The particle size of the nanoformulations is one of the crucial dependent factors for the optimization of NLC because it affected the stability and permeation of drugs across the layers of the tissues (Marwah et al., 2016). Therefore, in the fabrication of MZ-SO NLC, the impacts of all three employed independent variables were analyzed on the particle size of the nanoformulations. The particle size of the fabricated NLCs was found to be in the range of 92–410 nm (see Table 2), and this clearly showed the effects of the independent factors. The *F*-value of the model was 39.33, indicating the significance of the model (*p* < .05), and the difference between the predicted *R*^2^ value (0.8258) and adjusted *R*^2^ value (0.8778) was found to be less than 0.20 (Table 3). These results indicated reasonable agreement between the predicted and adjusted values of the particle size. In this obtained model, the interactions of independent factors on particle size were recorded as either a positive or negative effect on its quality attributes, and this was further analyzed by Equation (2) as follows:

(2) Particle size=+224.82+8.63 A−52.49 B−82.72 C …….

**Table 3.** Regression analysis results for selected responses (i.e. particle size, dissolution efficiency, and inhibition zone).

Responses	*R* ^2^	Adjusted *R*²	Predicted R²	Coefficient of variation (%CV)	Adequate precision
Particle size	0.9008	0.8778	0.8258	14.88	17.7249
Dissolution efficiency	0.9056	0.8838	0.8232	8.50	20.0078
Inhibition zone	0.9430	0.9298	0.8935	9.25	26.1972

**Figure 2.**
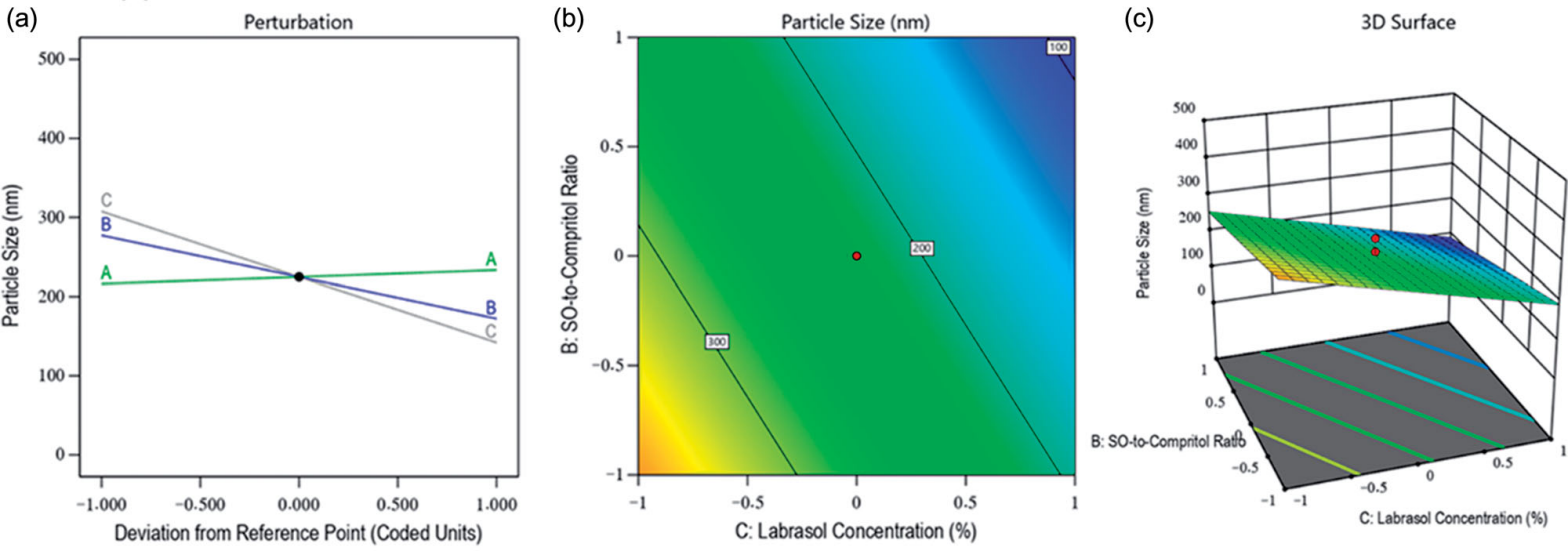
Effects of independent factors on the particle size of different prepared drug-loaded NLCs: (a) main effect plot, (b) contour plot, and (c) 3D surface plot.

Equation (2) indicated the positive impact of the total lipid-to-MZ ratio (+8.63 A) on particle size. It means the particle of the NLCs was increased with the increased concentration of factor A, and it was due to the maximum entrapment of drug in the lipids because of the lipophilic nature of MZ (Aljaeid & Hosny, 2016). In contrast, the SO-to-Compritol ratio and Labrasol concentration exhibited a negative impact on particle size. This meant that the size of the particles of the nanoformulations was decreased with the increased concentration of independent factors B and C. The reasons behind this phenomenon were found to be a better affinity of MZ toward these factors and the emulsification characteristics of Labrasol, which minimized the particle size and increased the stability of the nanoformulations (Imran et al., 2020). Simultaneously, the contour plot and 3D surface plot (Figure 2) exhibited the impact of the independent factors on the particle size of various fabricated NLCs.

#### 3.1.2. Impacts of independent factors on dissolution efficiency

The development of a nanoformulation with better dissolution efficiency was the aim of this study. Therefore, the dissolution efficiency was selected as a dependent factor for the optimization of NLC. As it is well-established that improved dissolution can also affect the solubility of a drug at diseased sites. So, in the fabrication of MZ-SO NLC, the impacts of all three of the independent variables were analyzed for the dissolution efficiency of the nanoformulations. The percentage of dissolution efficiency of various fabricated NLCs was in the range of 36–88% (see Table 2), and this clearly showed the effects of the independent factors. The F-value of the model was 41.55, indicating the significance of the model (*p* < .05), and the difference between the predicted *R*^2^ value (0.8232) and adjusted *R*^2^ value (0.8838) was less than 0.20 (see Table 3). These results indicated a reasonable agreement between the predicted and adjusted values of the percentage of dissolution efficiency. In this obtained model, the interactions of independent factors on the dissolution efficiency occurred as either a positive or negative effect on its quality attributes, and this was further analyzed by Equation (3) as follows:

(3) Dissolution efficiency=+57.35 − 9.03 A + 3.54 B + 11.09 C ……. 

**Figure 3.**
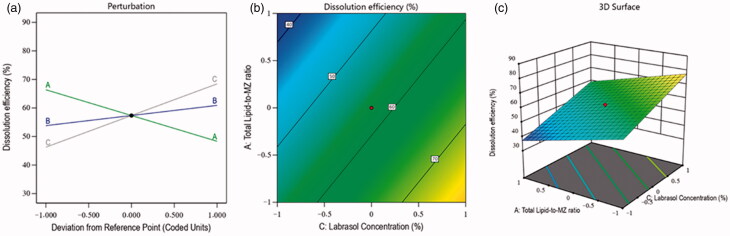
Effects of independent factors on the dissolution of different prepared drug-loaded NLCs: (a) main effect plot, (b) contour plot, and (c) 3D surface plot.

In this case, Equation (3) demonstrated a negative impact of the total lipid-to-MZ ratio (−9.03 A) on dissolution efficiency. It means, the dissolution of NLCs was decreased with the increased concentration of factor A, and this was due to the higher affinity of MZ for the lipid and the restricted solubility of the drug-entrapped lipid in the aqueous phase. In contrast, the SO-to-Compritol ratio and Labrasol concentration showed a positive effect on the dissolution efficiency of the nanoformulation. It means that the dissolution efficiency of the NLCs was increased with the increased concentration of independent factors B and C. The reasons behind this situation were found to be the emulsification characteristics of both factors A and B, which reduced the particle size and interfacial tension between the lipid and the aqueous phase and improved the dissolution efficiency of the prepared nanoformulations (Hosny et al., 2020). Simultaneously, the contour plot and 3D surface plot (Figure 3) exhibited the impacts of independent factors on the dissolution of various fabricated NLCs.

#### 3.1.3. Impacts of independent factors on inhibition zone

In order to evaluate the effect of selected independent factors on OCA, various NLC formulations were prepared. Since therapeutic efficacy is a detrimental factor in the treatment of candidiasis, therefore inhibition zone was selected as a parameter, and in the fabrication of MZ-SO NLC, the effects of selected independent factors were analyzed on the basis of inhibition zone. The effects were in the range of 5–29 mm (see Table 2). The F-value of the model was 71.69, indicating the significance of the model (*p* < .05), and the difference between the predicted *R*^2^ value (0.8935) and adjusted *R*^2^ value (0.9298) was less than 0.20 (see Table 3). These results indicated reasonable agreement between the predicted and adjusted values of the inhibition zone. In this model, the interactions of independent factors on the inhibition zone occurred as either a positive or negative effect on its quality attributes, and this was further analyzed by Equation (4) as follows:

(4) Inhibition zone=+16.88 − 2.13 A + 5.22 B + 2.57 C ……. 

**Figure 4.**
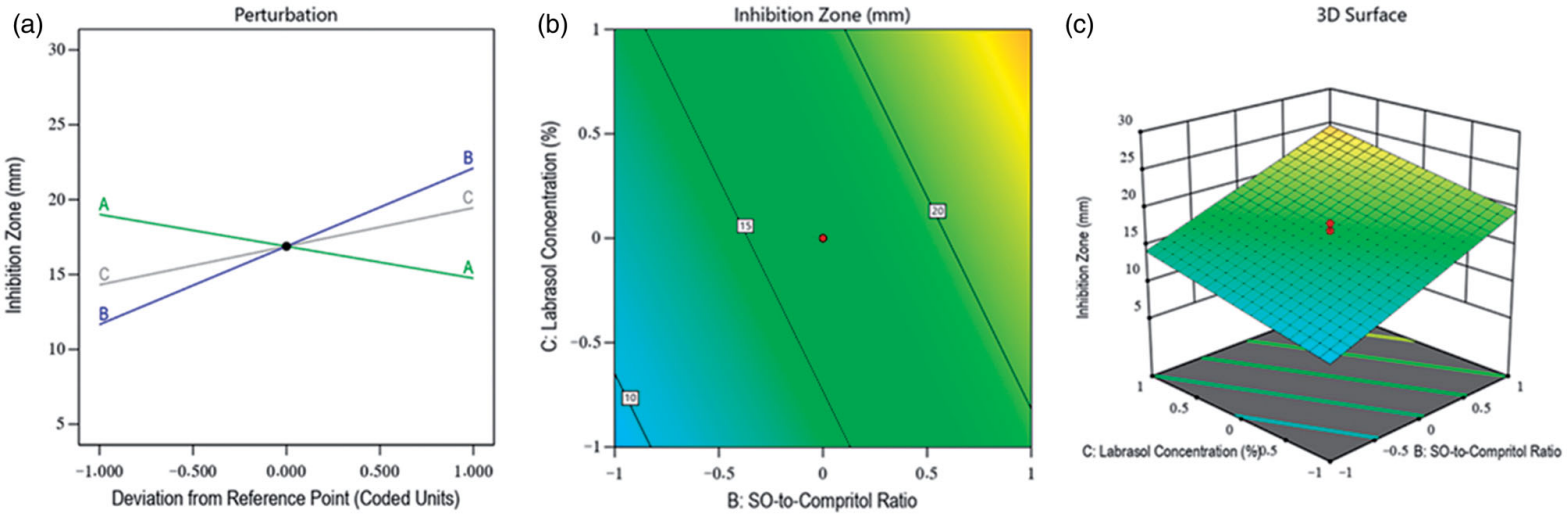
Effects of independent factors of different prepared drug-loaded NLCs on the inhibition zone: (a) main effect plot, (b) contour plot, and (c) 3D surface plot.

In this case, Equation (4) demonstrated the negative impact of the total lipid-to-MZ ratio (−2.13 A) on the inhibition zone. It means the inhibition zone was decreased with the increased concentration of factor A, and it was due to the limited effect of the lipid on the growth of fungus. Therefore, lipid concentrations were found unable to inhibit the growth of the fungus. The SO-to-Compritol ratio and Labrasol concentration in the prepared NLCs showed a positive effect on the inhibition zone. It means the SO-to-Compritol ratio and Labrasol concentration of the NLCs could restrict fungal growth in the selected medium with their increased concentrations. The reasons for this were the antifungal activity of SO (Lavaee et al., 2019) and the emulsification property of Labrasol, which increased the antifungal activity via the accelerating permeability (Alkhalidi et al., 2021) of MZ-SO-loaded NLCs. Additionally, the contour plot and 3D surface plot (Figure 4) exhibited the impacts of independent factors on the inhibition zone of various fabricated NLCs.

#### 3.1.4. Optimization of MZ-SO NLC

**Figure 5.**
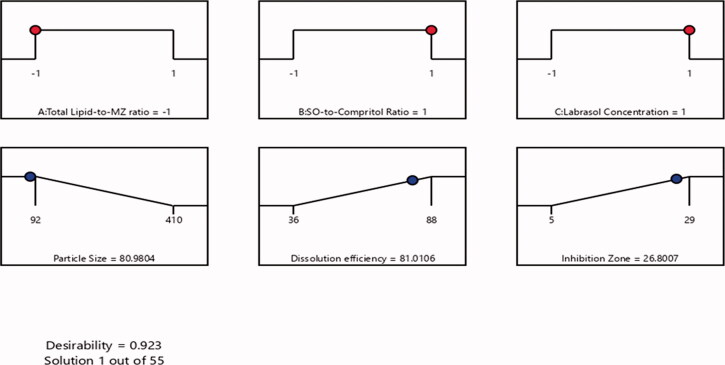
The desirability ramp shows the levels of the predicted and independent values for the optimized nanoformulation responses.

After characterization of all of the 17 runs suggested by the experimental design software on selected independent variables, an optimized MZ-SO NLC was obtained. The optimized formula contained a 10:1 ratio for total lipid to MZ, a 4:4 ratio for SO to Compritol, and a 2% Labrasol concentration. The optimized MZ-SO NLC had a particle size of 92 nm, zeta potential 23 ± 2 mV, polydispersability index of 0.183 ± 0.006, dissolution efficiency of 88%, and inhibition zone of 29 mm with 0.923 desirability (Figure 5). The desirability demonstrated the levels of the predicted and independent values of the procured responses of the optimized NLC. Table 4 demonstrated a close agreement of the predicted and experimental data of the optimized MZ-SO NLC, and it showed a nonsignificant difference (*p* > .05) between the data. This affirmed the exactness of the equation and its validity.

**Table 4.** Predicted and experimental responses of the optimized nanoformulation.

Solution	Total lipid-to-MZ ratio	SO-to-compritol ratio	Labrasol (%)	Particle size (nm)	Dissolution efficiency (%)	Inhibition zone (mm)	Desirability
Predicted value	10:1	4:4	2	80.98	81.01	26.80	0.923
Experimental value	10:1	4:4	2	82	84	28	0.923

### 3.2. Ultramorphological analysis of MZ-SO NLC

**Figure 6.**
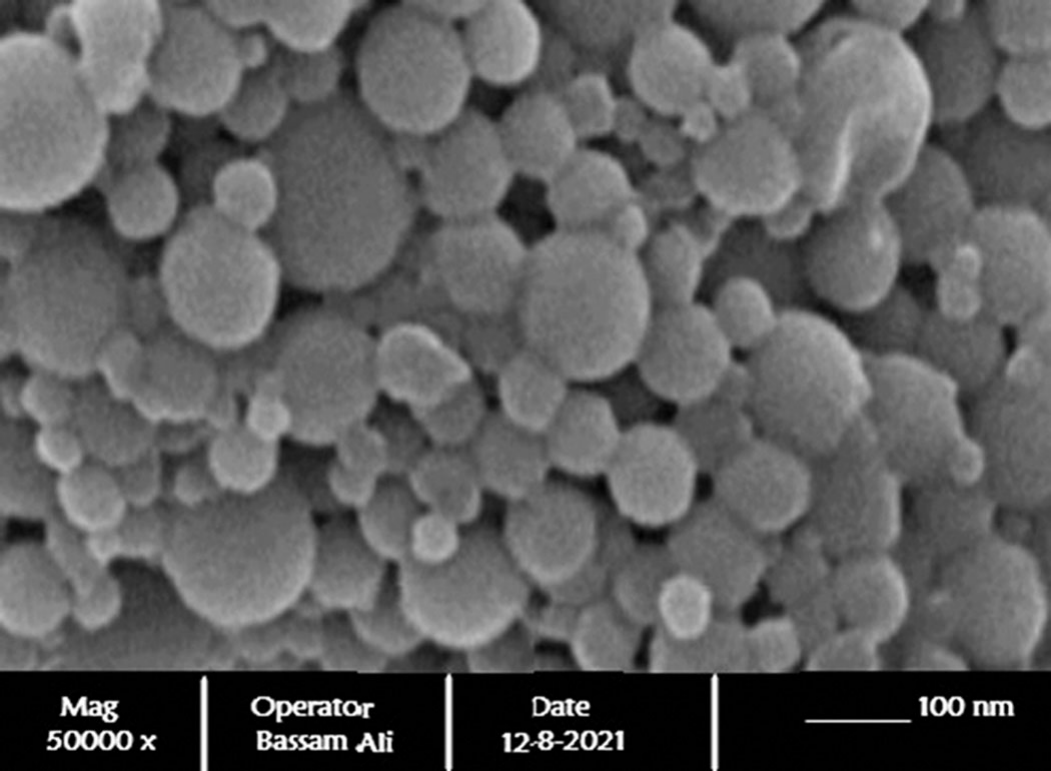
SEM of ultramorphological study of the optimized nanoformulation.

The surface morphology of the optimized MZ-SO NLC was evaluated by structural equation modeling (SEM). The photomicrograph (Figure 6) clearly demonstrated the spherical shape of the nanostructures with a size range of 70–110 nm. The SEM of the optimized NLC also in line with the results of the particle size obtained with the Zetatrac particle size analyzer.

### 3.3. *Ex vivo* permeation studies

A comparative *ex vivo* permeation study was conducted on the MZ-SO NLC, marketed MZ cream, and MZ aqueous suspension. This study hoped to find an improved permeability profile of the optimized MZ-SO NLC for better passive targeted efficacy against OCA. The cumulative amounts of MZ that permeated the buccal mucosa in the optimized MZ-SO NLC, marketed MZ cream, and MZ aqueous suspension was 1472 μg/cm^2^, 1215 μg/cm^2^, and 470 μg/cm^2^, respectively. Additionally, the steady-state flux, permeability coefficient, diffusion coefficient, relative permeation rate, and enhancement factor for the optimized NLC were found to be better than for the marketed MZ cream and MZ aqueous suspension (Table 5). The study outcomes showed a significant (*p* < .05) improved permeability for the optimized MZ-SO NLC compared with the marketed MZ cream and MZ aqueous suspension. Thus, the finding of this study proved that the optimized MZ-SO NLC is an effective tool for treating OCA. The rationale behind these improved findings was an occlusive effect of NLC, which alters the pores and accelerates permeation of the nanoformulation; additionally, the emulsifier in the optimized nanoformulation promoted permeation. The nanosize of the MZ-SO NLC was a pronounced and detrimental factor for improving the permeation of the drug via paracellular and transcellular routes (Marwah et al., 2016; Choudhury et al., 2019). Thus, the combination of the aforementioned factors improved the buccal permeation of MZ and enhanced the therapeutic efficacy of the drug via upgraded drug deposition.

**Table 5.** Determined permeation parameters for MZ from different formulations (*n* = 3; mean ± SD).

Permeation Parameters	Marketed MZ cream (Daktarin 2%, Janssen, India)	Optimized MZ-SO NLC formulation	MZ aqueous suspension
Cumulative amount permeated (μg/cm^2^)	1215 ± 22.27	1472 ± 20.42	470 ± 7.00
Steady state flux, Jss, (μg/cm^2^.min)	4.31 ± 0.15	5.61 ± 0.18	2.00 ± 0.17
Permeability coefficient, P, (cm/min)	1.01 ± 0.04 × 10^−4^	1.32 ± 0.13 × 10^−4^	0.51 ± 0.02 × 10^−4^
Diffusion coefficient, D, (cm^2^/min)	28.6 ± 3.25 × 10^−5^	37.2 ± 2.00 × 10^−5^	6.62 ± 0.77 × 10^−5^
Relative permeation rate (RPR)		1.21 ± 0.20	0.39 ± 0.03
Enhancement factor (F_en_)	2.59 ± 0.14	3.13 ± 0.21	

### 3.4. Determination of in vivo efficacy of optimized MZ-SO NLC against OCA

In order to determine the *in vivo* efficacy, the animals of different groups were treated with their corresponding formulation using a sterile cotton swab, and findings of the study are depicted in Table 6. As shown, the use of the optimized 2% MZ-SO NLC significantly reduced the ulcer index compared with the commercially available 2% MZ marketed oral gel, SO alone, or normal saline-treated group. Additionally, SO alone showed an improved antiulcer effect compared with the commercially available 2% MZ marketed oral gel but was found to be inferior to the optimized 2% MZ-SO NLC. Thus, the findings revealed the additive anti-ulcer effects of SO, which improved the therapeutic value of MZ in a nanoformulation.

**Table 6.** Ulcer index values of different animal groups (mean ± SD, *n* = 6).

Animal group	Ulcer index
optimized 2% MZ-SO NLC	0.5 ± 0.50
SO alone	1.5 ± 0.25
Commercially available 2% MZ marketed oral gel	2.5 ± 0.25
Normal saline	4 ± 0.5

## 4. Conclusion

The optimal MZ-SO NLC formulation was successfully developed using a CCD-based experimental design. Different NLCs were prepared and characterized for particle size, dissolution efficiency, and inhibition zone. For the optimized MZ-SO NLC, the experimental design software demonstrated a 0.923 desirability, which indicated a close agreement between the predicted and experimental data in terms of the declared exactness of the equation and validity. A photomicrograph of the ultramorphological analysis demonstrated the spherical shape of the nanostructures that further signifies the success of the formulation. The permeation efficiency of the optimized MZ-SO NLC was determined using sheep buccal mucosa via a Franz diffusion cell. The findings of the permeation study showed that the permeation of the nanoformulation was enhanced as compared to the marketed MZ formulation and aqueous MZ suspension. An *in vivo* antifungal study in terms of the ulcer index showed results in favor of the optimized MZ-SO NLC. Hence, it can be concluded that when MZ is applied in an optimized NLC of SO, it can effectively treat candidiasis by inhibiting the growth of CA.

## Data Availability

Data used to support the findings of this study are included within the article.
